# Supplementary material for: FAIR assessment of Disease Maps fosters open science and scientific crowdsourcing in systems biomedicine
Source: Sci Data. 2025 May 23;12:851. doi: 10.1038/s41597-025-05147-w (PMC12102143; doi:10.1038/s41597-025-05147-w)
Supplement: Supplementary file 1 — FAIR assessment of the MINERVA platform using the FAIR Software checklist (https://fairsoftwarechecklist.net/v0.2/) [file 41597_2025_5147_MOESM1_ESM.docx]

# **FAIR assessment of the MINERVA platform using the FAIR Software checklist (**[**https://fairsoftwarechecklist.net/v0.2/**](https://fairsoftwarechecklist.net/v0.2/)**)**

Checklist results available here: <https://fairsoftwarechecklist.net/v0.2?f=31&a=32112&i=32322&r=133>.

## Findable

1. Is information from the metadata consumed by search engines to help rank their results? For the definition of "the metadata", see section above.

**Yes, the software is findable via an associated web page which employs Search Engine Optimization.**Explanation: the MINERVA Platform is featured on bio.tools (<https://bio.tools/MINERVA_Platform>).

2. Are the software's identifiers globally unique? For the definition of "the software", see section above.

**Yes for at least some of the identifiers.**

Explanation: URLs uniquely pointing to the MINERVA Platform are <https://minerva.uni.lu> and <https://bio.tools/MINERVA_Platform>.

## Accessible

3. Can someone else use the software's identifiers to get a copy of the software?

**Yes for at least some of the identifiers.**

Explanation: Materials at <https://minerva.uni.lu> explain how to set up MINERVA Platform.

4. A few years from now, will the software's identifiers resolve to exactly the same content as they do today?

**Yes, as previous answer, and additionally the metadata will be available for some time after the software itself is no longer available.**

Explanation: The website <https://bio.tools/MINERVA_Platform> is unique and will be persisted. Moreover, the MINERVA Platform is a part of ELIXIR Luxembourg service portfolio and its sustainability strategy (see <https://elixir-europe.org/about-us/who-we-are/nodes/luxembourg>).

5. Is the software retrievable using a protocol that is open, free, and universally implementable?

**Yes**

Explanation: Software is available on GitLab.

6. Is the software retrievable using a protocol that allows for authentication and authorization?

**Yes, or the software is openly available.**

Explanation: The MINERVA Platform is openly available.

7. Does the metadata include an identifier to reference the associated software?

**Yes, the metadata includes a unique identifier to reference the software.**

Explanation: The metadata (i.e. <https://bio.tools/MINERVA_Platform>, <https://minerva.pages.uni.lu>) refers to the GitLab repository.

## Interoperable

8. If the software reads, writes, or exchanges data, what type of formats are being used?

**As previous answer, and additionally the formats are common for the domain.**

Explanation: The MINERVA Platform handles major community formats for systems biology diagrams (SBGN, SBML layout+render, GPML).

9. What approach does the software take to versioning?

**As previous answer, and additionally, the version identifier communicates information about compatibility.**

Explanation: In the implemented versioning system, major versions are not backwards compatible (<https://minerva.pages.uni.lu/doc/releasenotes/>).

10. How are the software's dependencies specified?

**As previous answer, and additionally each link points to a specific version or version range of the dependency, and the list of dependencies includes all dependencies needed to get the software running again on a fresh install of the targeted operating systems.**

Explanation: Each version of the MINERVA Platform is released as a dedicated debian package, specifying dependencies and allowing fresh install.

11. How is the software's relationship to related software, other than its dependencies, described?

**As previous answer, with at least some of the links qualified and machine-readable.**

Explanation: bio.tools profile of the MINERVA Platform indicates qualified relationships to software available in the bio.tools platform.

12. How is the software's relationship to related data described?

**As previous answer, with at least some of the links qualified and machine-readable.**

Explanation: the documentation of the MINERVA Platform describes details of handled data formats, and annotations of element and interactions in systems biology diagrams.

## Reusable

13. Is the software executable?

**Yes, the software can be compiled using information bundled with the software sources.**

Explanation: debian packages are available for each released version. Compilation from sources is also possible and documented.

14. Is the software made available in a form that allows for it to be understood, modified, built upon, or incorporated into other software?

**Yes, as previous answer, and additionally the software is available via a package manager.**

Explanation: the code on GitLab is documented, the MINERVA platform is available via debian package manager.

15. Which of the following best describes the software's usage rights?

**As previous answer, and additionally, the license is machine-readable.**

Explanation: information is available in the LICENSE file in the GitLab repo.
